# Impaired Proteostasis Contributes to Renal Tubular Dysgenesis

**DOI:** 10.1371/journal.pone.0020854

**Published:** 2011-06-09

**Authors:** Rita Machado de Oliveira, Zrinka Marijanovic, Filipe Carvalho, Gabriel Miltenberger Miltényi, Joana Estevão Matos, Sandra Tenreiro, Sónia Oliveira, Francisco Javier Enguita, Rosário Stone, Tiago Fleming Outeiro

**Affiliations:** 1 Cell and Molecular Neuroscience Unit, Instituto de Medicina Molecular, Lisboa, Portugal; 2 GenoMed Diagnosticos de Medicina Molecular, Instituto de Medicina Molecular, Lisboa, Portugal; 3 Cell Biology Unit, Instituto de Medicina Molecular, Lisboa, Portugal; 4 Unidade de Nefrologia, Serviço de Pediatria, Hospital da Santa Maria, Lisboa, Portugal; 5 Instituto de Fisiologia, Faculdade de Medicina de Lisboa, Lisboa, Portugal; Consejo Superior de Investigaciones Cientificas, Spain

## Abstract

Protein conformational disorders are associated with the appearance, persistence, accumulation, and misprocessing of aberrant proteins in the cell. The etiology of renal tubular dysgenesis (RTD) is linked to mutations in the angiotensin-converting enzyme (ACE). Here, we report the identification of a novel ACE mutation (Q1069R) in an RTD patient. ACE Q1069R is found sequestered in the endoplasmic reticulum and is also subject to increased proteasomal degradation, preventing its transport to the cell surface and extracellular fluids. Modulation of cellular proteostasis by temperature shift causes an extension in the processing time and trafficking of ACE Q1069R resulting in partial rescue of the protein processing defect and an increase in plasma membrane levels. In addition, we found that temperature shifting causes the ACE Q1069R protein to be secreted in an active state, suggesting that the mutation does not affect the enzyme's catalytic properties.

## Introduction

A growing number of human diseases, such as cystic fibrosis, Alzheimer's disease, and certain types of cancer, are associated with alterations in the protein homeostasis network (proteostasis) that lead to protein misfolding, mislocalization, or aggregation [1].

RTD is a severe disorder affecting renal tubular development and is characterized by persistent fetal anuria and perinatal death [2]. Mutations in different components of the renin-angiotensin system have been linked to RTD, and one such class of mutations are those found in the gene that codes for the angiotensin-converting enzyme (ACE) [3]. In most cases, affected individuals die *in utero* or within 24 hours of birth [2]. ACE is a zinc-metallopeptidase and a key component of the renin-angiotensin-aldosterone system involved in the regulation of blood pressure and heart function through the formation of the vasoconstrictor angiotensin II and inactivation of the vasodilator bradykinin [4], [5]. ACE also regulates water balance, neuropeptide metabolism, reproduction, immune functions and kidney development [6], [7], [8], [9]. There are two forms of ACE, a somatic and a testicular form, both C-terminally anchored to the plasma membrane [10]. The somatic form is abundant in endothelial, epithelial and neuronal cell membranes. Somatic ACE also exists as a soluble form that originates from membrane-bound endothelial ACE by the action of a yet unidentified protease. Soluble ACE is found in the plasma, cerebrospinal fluid, seminal liquid, and urine [10], [11], and contains two enzymatic domains with a high degree of internal sequence homology [12]. However, the active sites in the two enzymatic domains display contrasting catalytic and immunological properties and substrate specificities [13], [14], [15].

Recently, two novel homozygous mutations in the ACE gene linked to autosomal recessive RTD were described [16], [17]. Here, we describe a novel point mutation in the ACE gene, encoding ACE Q1069R, identified in a female RTD patient. We elucidated the molecular mechanisms by which this mutation results in non-functional ACE protein and discuss possible strategies for therapeutic intervention in RTD and other disorders associated with proteostasis network imbalance.

## Results

### Identification of the mutation in the ACE gene

In 2004, a female child was born by cesarean section after 36 weeks of gestation. Apgar score was 2 at the fist minute and 8 at the tenth minute after birth. Somatometrics was adequate to the gestational age. There was no history of parental consanguinity and no reported cases of renal disease in the family. The mother was a healthy young women and the pregnancy was uneventful, with no reference to oligoamnious or to maternal drugs use. The patient had a healthy older brother.

At birth, the patient presented large fontanels with widely separated sutures, talus feet and joint contractures. Profound hypotension recalcitrant to treatment with pressors was present since the first hours after birth. She also presented moderate respiratory distress and a persistent ductus arteriosus. Anuria was detected at birth so peritoneal dialysis was started on the 3^rd^ day after birth. The renal sonogram showed normal/high size kidneys, with poor corticomedullary differentiation. The skull x-ray showed poor ossification of the vault. Biochemical investigation indicated very high plasmatic rennin (>1000 mcU/ml) and the karyotype was normal (46 XX). Based on the above symptoms clinical diagnosis of RTD was suspected. On the 12^th^ day pressor therapy was stopped and she was transferred to the nephrology unit where she was treated as an inpatient on continuous peritoneal dialysis for 6 months. At that age, the patient started cycling peritoneal dialysis and was discharged from the hospital. Diuresis slowly increased until 1.5 ml/kg/day and the main problem was failure to thrive with slight developmental retardation. At the age of 4 years the patient was successfully submitted to cadaver kidney transplantation. She maintains normal graft function, improved growth and normalized development.

### Identification of the mutation in the ACE gene

**Figure 1:**
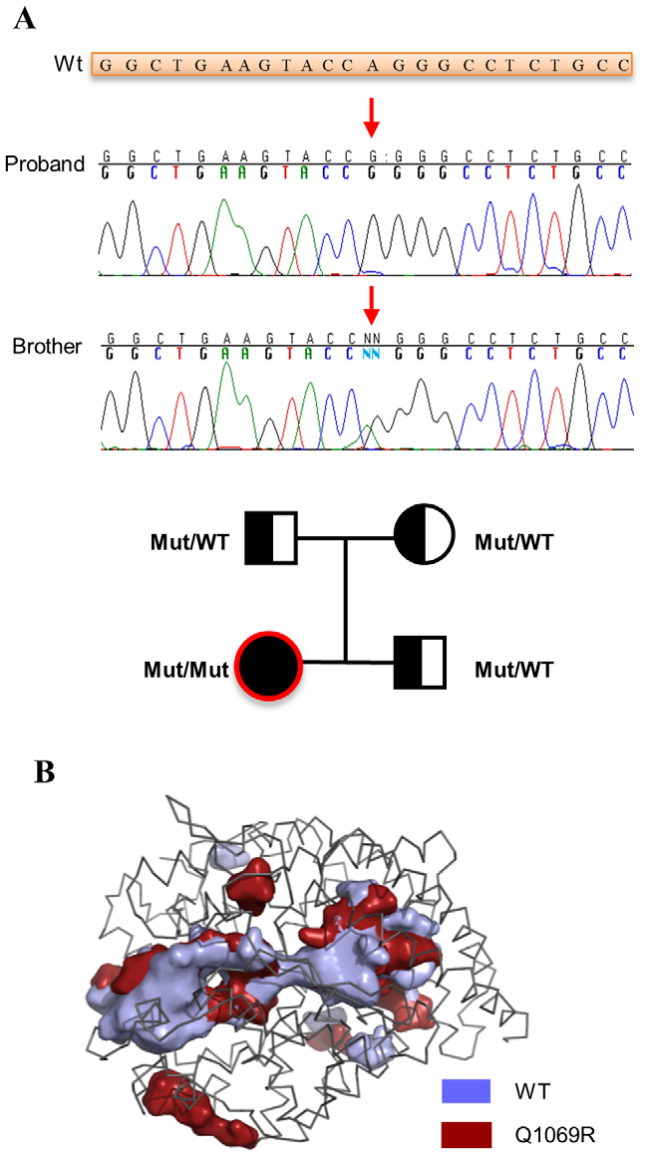
Novel mutation in the ACE gene is associated with RTD in a homozygous patient for the nucleotide substitution. **A.** Mutation c.3293A>G (p.G1069R) in homozygous state in our proband and in heterozygous state in the patient’s brother. Arrow points the changing nucleotide. Wt: WT sequence of the ACE gene (part of exon 22). On the bottom, pedigree of the family. Circles indicate females; squares, males; filled symbols, affected individuals; open symbols, unaffected individuals; Mut/Mut, homozygous p.Q1069R mutation; Mut/WT, heterozygous Q1069R mutation (WT indicates WT allele). **B.** Pocket and cavity analysis of C-terminal domains from native ACE and Q1069R mutant after energy minimization of the atomic coordinates and molecular dynamics simulations. Presence of pocket and cavities within the structure were analyzed by POCASA algorithm and the results plotted in 3D structure over the backbone of the native ACE C-terminal domain atomic coordinates (PDB code: 2OC2). The figure was prepared by using Pymol.

Since ACE was previously implicated in RTD, we sequenced the ACE gene from our patient and identified a homozygous A-to-G nucleotide change in exon 22 (c.3293A>G) (Figure 1A). The mutation caused a glutamine to arginine substitution at amino acid 1069 in the ACE protein. Segregation analysis in the family of the proband showed that both of her clinically healthy parents and one clinically healthy brother were heterozygous carriers for this mutation. No other mutations were observed in the ACE gene (Figure 1A).

### Impact of the mutation on protein structure and activity

We assayed ACE activity from patient blood samples to determine whether the mutation affected its enzymatic activity. In contrast to the reported absence of activity prior to kidney transplant [18], the patient displayed normal ACE activity compared to five healthy volunteers (Figure S1). Lack of ACE activity from the patient sample prior to kidney transplantation may stem from changes in the active site of the mutant protein or alternatively, the mutation may affect protein localization and stability. In order to distinguish between these two possibilities we employed molecular dynamics simulations of wild type (WT) and mutant protein in a controlled environment. See SI for more details about the molecular dynamics simulation. Root Mean Squared Deviations (RMSD) of the amino-acids positions of WT and mutant proteins, along with the simulation of the 3-D structure, suggests that the Q1069R substitution should not influence the structure of the active site (Figure 1B and Figure S2). Amino acids comprising the active site of the enzyme and residues involved in coordinating a Zn^2+^ atom required for protein activity showed little or no variation in RMSD in each protein, suggesting that lack of activity is likely due to aberrant protein processing (Figure S2). Moreover, molecular dynamics analysis showed an increased exposed surface area in the mutated protein caused by changes in the protein hydrodynamic radius (Figure S2), consistent with an increase in the volume of internal pockets and cavities present in the mutant protein relative to the WT protein (Figure 1B).

### Stability and cellular localization of the mutant ACE protein

**Figure 2:**
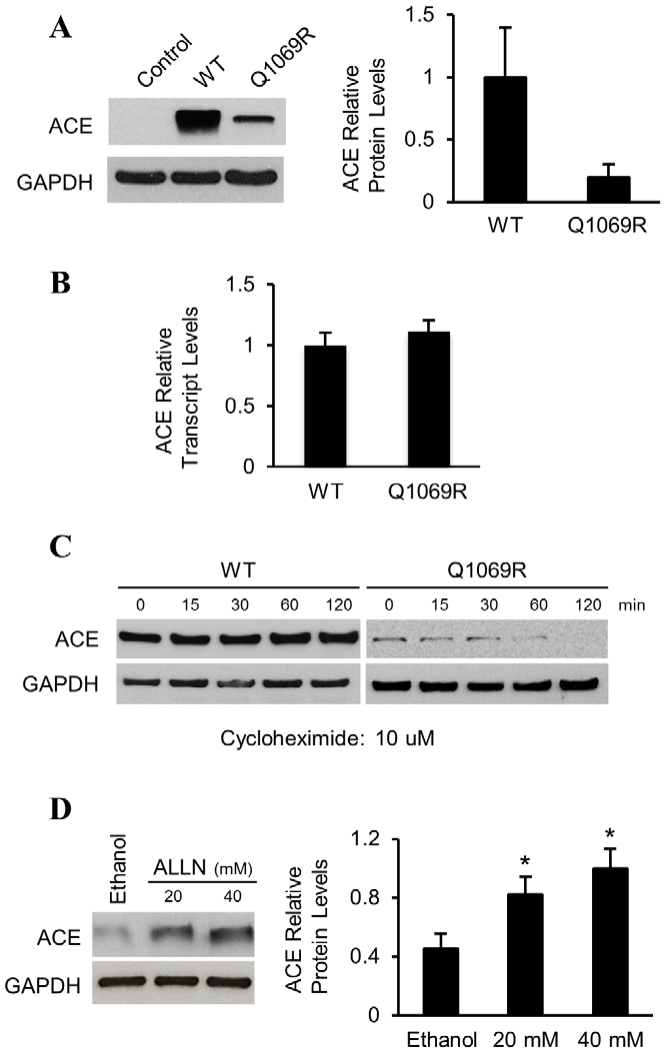
Low ACE_Q1069R_ protein levels in HEK cells is due to degradation by the ubiquitin proteasome system. **A.** Analysis of ACE protein levels by western blot in HEK cells stably expressing the WT form or the mutant (Q1069R). Empty vector is used as a negative control. GAPDH was used as a loading control. Quantification of 3 independent experiments is shown on the right. Error bars represent ±SD. P<0.05. **B.** Relative quantitative analysis of ACE mRNA levels in HEK cells stably expressing WT or ACE_Q1069R_, normalized to β-actin. Error bar represents ±SD. **C.** Analysis of ACE protein stability by pulse-chase experiment in HEK cells. Cells were treated with CHX, for the indicated times. Protein levels were assessed by western blot analysis. Quantification of 3 independent experiments is shown. Error bars represent ±SD. P<0.05. **D.** Western blot analysis of ACE_Q1069R_ HEK cells. Cells in growth medium were treated for 2h with control medium or media with increasing dosages of ALLN. On the right, quantification of three independent experiments is shown. Error bars represent ±SD. P<0.05.

In order to study ACE processing, we established a cellular model to analyze localization, trafficking and turnover of WT and mutant ACE. We generated stable cell lines expressing either WT ACE (ACE_WT_) or ACE Q1069R (ACE_Q1069R_) in a human embryonic kidney (HEK) cells. We performed immunoblot analysis to investigate ACE protein levels both cell lines using an anti-ACE antibody that recognizes the denatured form of the protein (anti-ACE 1D8). ACE protein levels were approximately 80% lower in ACE_Q1068R_ cells compared to ACE_WT_ cells (Figure 2A); the lower level of mutant ACE protein in ACE_Q1068R_ cells could be attributed to lower protein expression, decreased protein stability, or both. To discriminate between these possibilities, we performed quantitative RT-PCR analysis on ACE_WT_ and ACE_Q1069R_ cells quantifying ACE mRNA levels and found no significant difference between mRNA levels (Figure 2B). Thus, the marked discrepancy between the mRNA and protein levels of wild type and mutant ACE-expressing cells suggested reduced protein stability for the mutant form. Next, we analyzed the half-life of ACE_WT_ and ACE_Q1069R_. Cells were treated with cyclohexamide (CHX) to inhibit *de novo* protein synthesis and protein levels were monitored by immunoblot analysis. While the ACE_WT_ protein showed essentially no decrease in protein levels over a 120 minutes time course, ACE_Q1069R_ protein is more rapidly degraded and is not detected at 120 minutes, suggesting a decreased stability of the mutant protein (Figure 2C).

To test whether the degradation of ACE_Q1069R_ protein occurs via the ubiquitin/proteasome system (UPS), we performed immunoblot analysis in the presence of increasing concentrations of the proteasome inhibitor ALLN. We observed a dose-dependent increase in the level of mutant ACE protein suggesting that the mutant form was degraded by the proteasome (Figure 2D). These results indicate that the lower level of ACE_Q1069R_ is due to selective degradation of the mutant form by the UPS.

**Figure 3:**
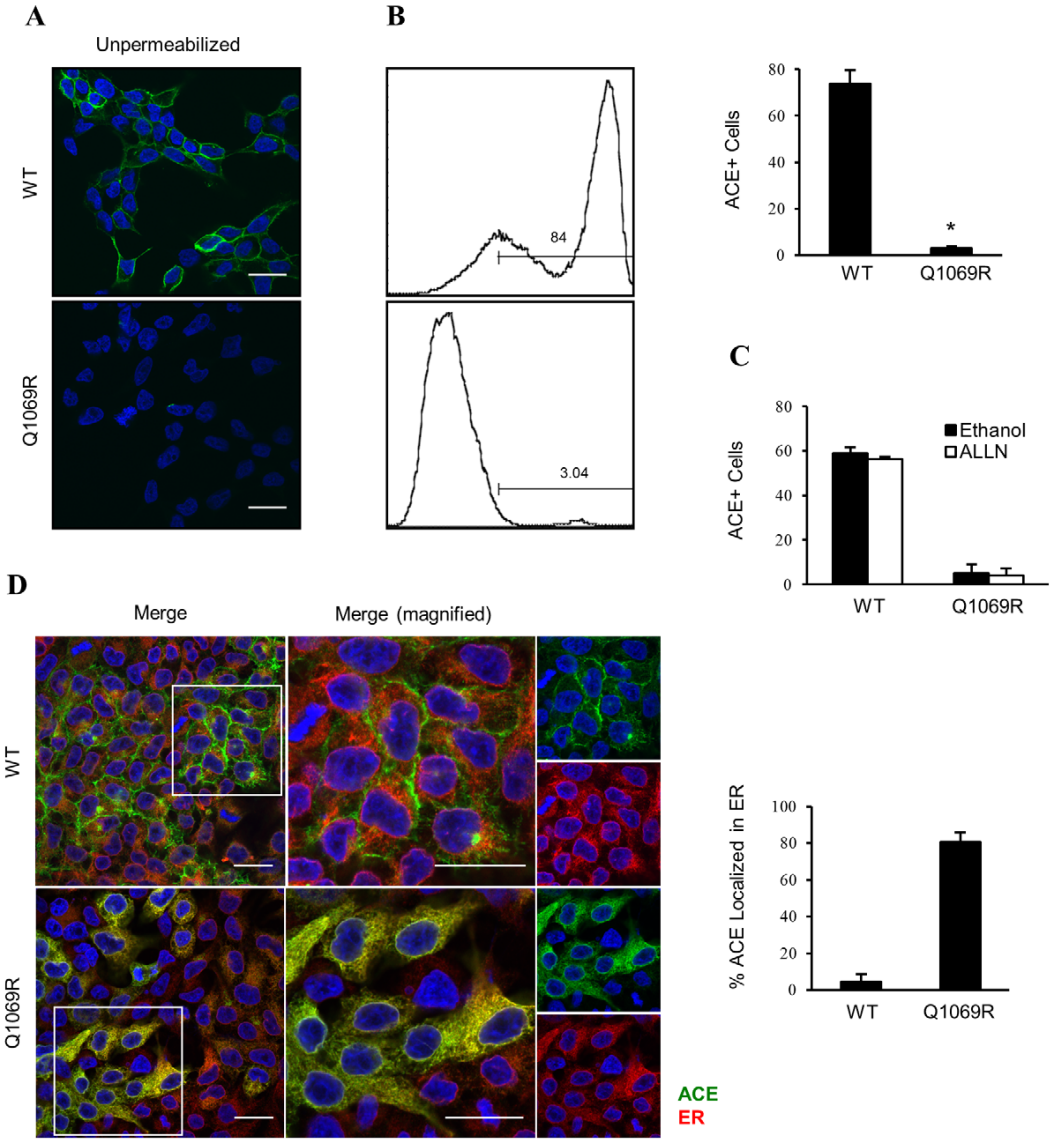
ACE_Q1069R_ protein is sequestered intracellular and does not reach the plasma membrane. **A.** HEK cells plated on glass cover slips for 2 days then fixed and not permeabilized and immunostained for ACE (green). Immunofluorescence staining observed by confocal microscopy. Scale bars, 20 µm. **B.** Analysis of ACE protein by flow cytometry in non-permeabilized ACE_WT_ and ACE_Q1069R_ stable cells. Quantification of 3 independent experiments is shown. Error bars represent ±SD. P<0.05. **C.** Analysis of ACE protein by flow cytometry in non-permeabilized ACE_WT_ and ACE_Q1069R_ stable cells grown in the presence of ALLN or control media for 2h. Quantification of 3 independent experiments. Error bars represent ±SD. **D.** HEK cells plated on glass cover slips for 2 days then fixed and immunostained for ACE (green), ER (red) and nuclei are stained blue with DAPI). Immunofluorescence staining observed with confocal microscopy. Insets represent enlarged images in the boxed regions. Scale bars, 20 µm. Quantification of the green signal overlapping with red signal was done using ImageJ software.

In order to investigate ACE's cell surface localization, we performed immunofluorescence analysis on both stable cell lines using an antibody that recognizes the native form of the protein (anti-ACE i2H5). Using non-permeabilizing conditions, we found a well-defined membrane pattern in ACE_WT_ cells, while ACE_Q1069R_ cells failed to show ACE protein on the cell surface (Figure 3A). These results were confirmed by flow cytometry (Figure 3B).

To assess whether the absence of plasma membrane localization of the mutant protein was due to overall lower protein levels and/or impairment in protein trafficking, the ACE_Q1069R_ stable cell line was treated with proteasome inhibitor and surface levels of ACE were analyzed by flow cytometry and epifluorescence microscopy. Although we detected an increase in ACE_Q1069R_ protein level in the presence of ALLN we could not observe any increase in the level of the protein at the cell surface (Figure 3C), suggesting that impaired protein trafficking caused the absence of ACE_Q1069R_ at the cell surface. Next, we analyzed the subcellular localization of ACE_WT_ and ACE_Q1069R_ protein in stable cell lines by epifluorescence microscopy. Immunofluorescence analysis using the organelle markers calnexin, for the ER, and giantin, for the Golgi, showed that approximately 80% of ACE_Q1069R_ was found in the ER, while only 10% of the WT protein was found in the same compartment (Figure 3D). Furthermore, approximately 30% of WT and 10% of mutant protein were found in the Golgi, implying impaired ER to Golgi trafficking of ACE_Q1069R_ (Figure S3). Taken together, these results suggest that mutant ACE is not able to reach the plasma membrane, accumulates in the ER and, as a consequence, is rapidly degraded by the proteasome. The sequestration of ACE_Q1069R_ in the ER suggests that it might be misfolded.

**Figure 4:**
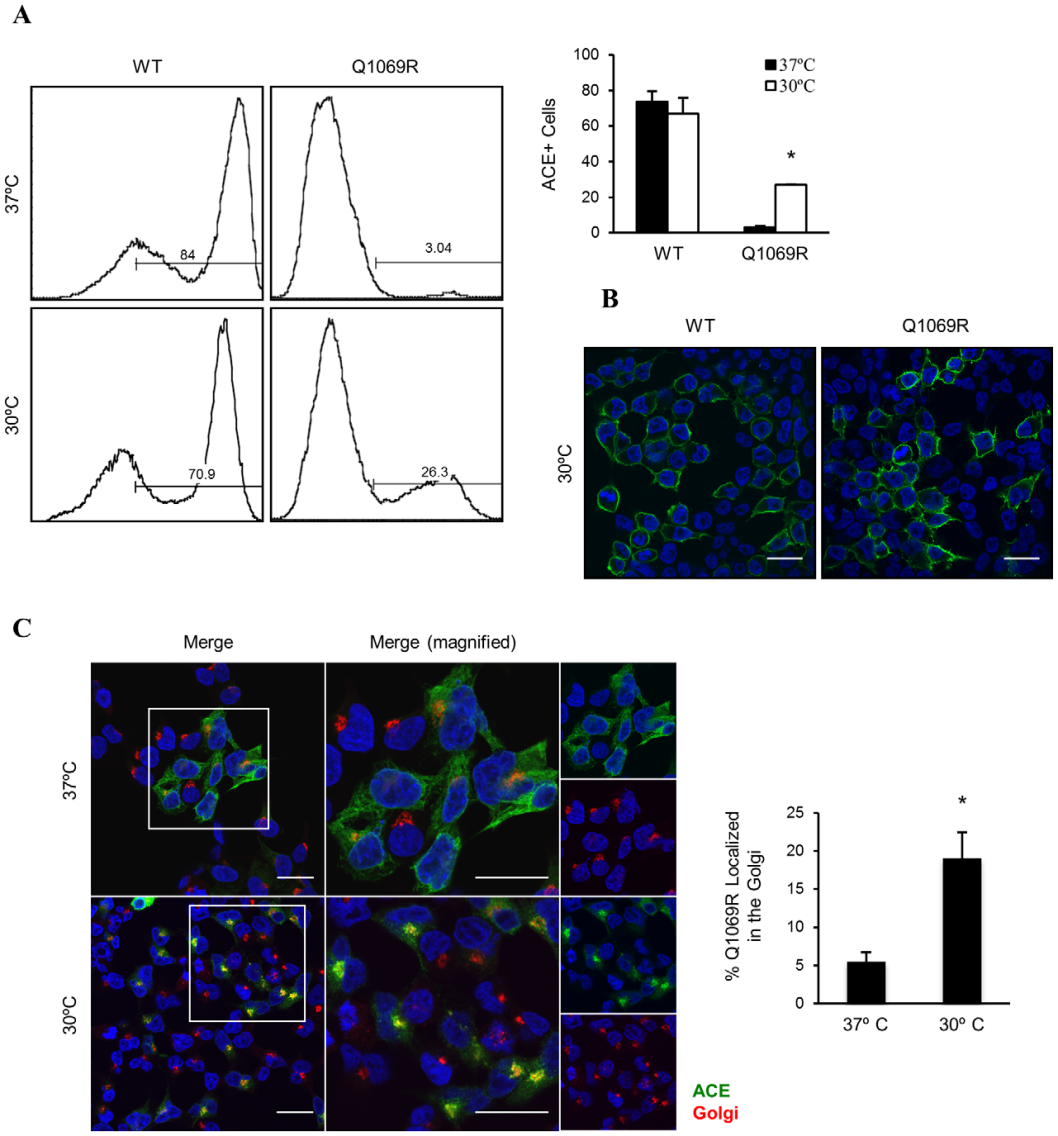
ACE_Q1069R_ in the plasma membrane at 30°C due to an improvement of trafficking through the secretory pathway. **A.** Flow cytometry analysis of ACE protein levels at the plasma membrane in non-permeabilized cells. ACE_Q1069R_ and ACE_WT_ HEK cells grown at 37°C or 30°C for 4 days. Quantification of 3 independent experiments. Error bars represent ±SD. P<0.05. **B.** Immunostain analysis by confocal microscopy of ACE_Q1069R_ HEK cells grown in glass coverslips at 37°C versus 30°C for 4 days. Cell not permeabilized and fixed were stained with ACE in green, nuclei are stained blue with DAPI. Scale bars, 20 µm. **C.** Same conditions and cells as in A. Cells were permeabilized and double stained for Golgi (red) and ACE (green). Nuclei are stained blue with DAPI. Insets represent enlarged images in the boxed regions. Scale bars, 20 µm. Quantification of the green signal overlapping with red signal was done using ImageJ software.

**Figure 5:**
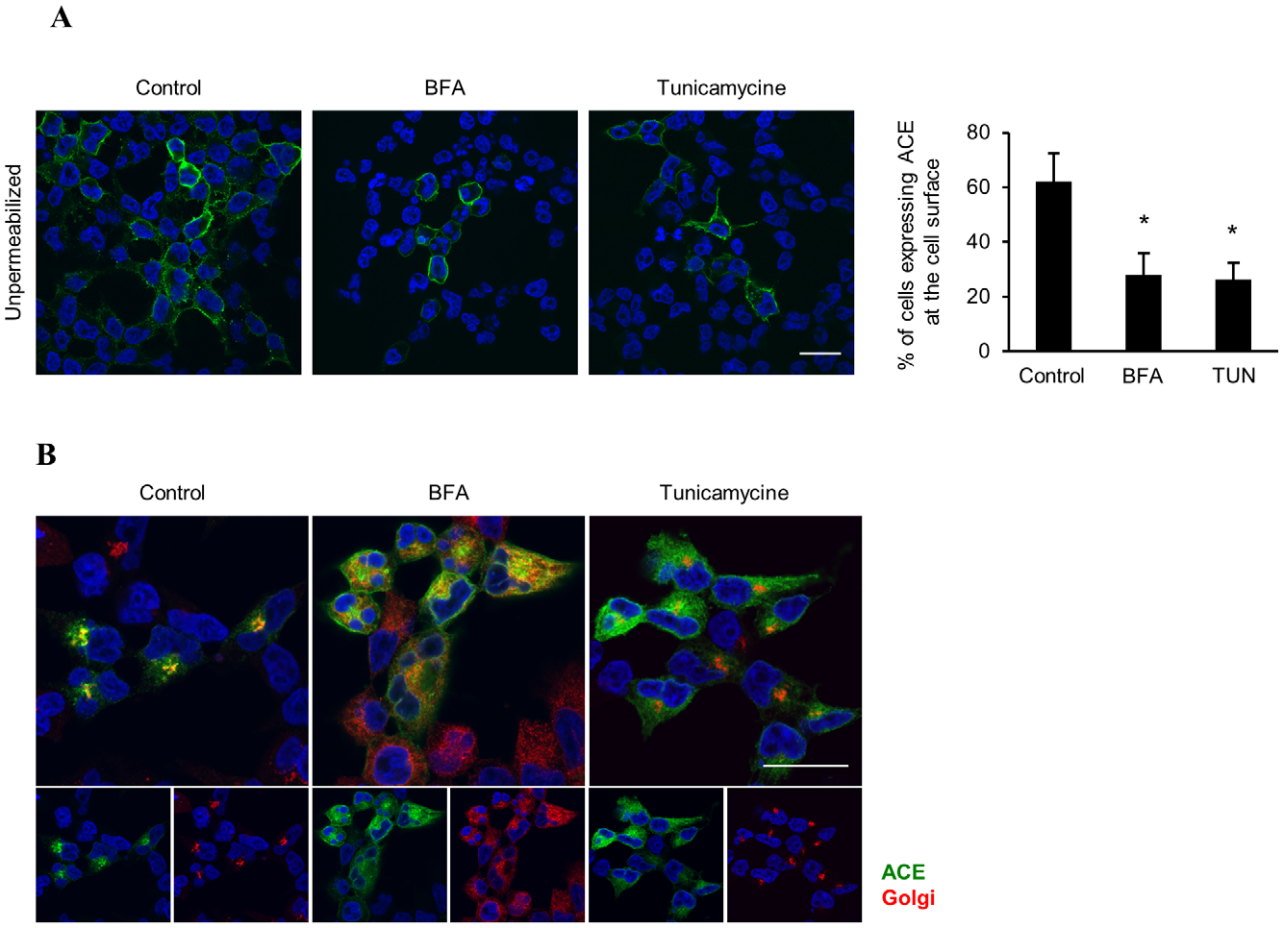
BFA and tunycamicin abolishes the 30°C rescue of ACE_Q1069R_ at the plasma membrane. **A.** Immunostaining analysis by confocal microscopy of ACE_Q1069R_ HEK cells grown at 30°C on glass coverslips in the presence of 10 µg/ml Tunycamicin for 6h, 10 µg/ml BFA for 6h or control media. Cells were not permeabilized and stained with ACE (green). Scale bars, 20 µm. Quantification of number of cell which display ACE membrane signal. Quantification of 3 independent experiments. Error bars represent ±SD. **B.** Immunostain analysis by confocal microscopy of ACE_Q1069R_ HEK cells grown in the same conditions as in A. Cells were fixed, permeabilized, stained for ACE (green) and Golgi (red). Nuclei are stained blue with DAPI. Insets represent enlarged images in the boxed regions. Scale bars, 20 µm. Quantification of the green signal overlapping with red signal was done using ImageJ software. Quantification of 3 independent experiments. Erros bars represent ±SD.

Incubation of cells at 30°C increases the interaction of misfolded proteins with molecular chaperones, facilitating their proper folding and trafficking. Thus, we analyzed the subcellular localization of ACE_WT_ and ACE_Q1069R_ by flow cytometry using the i2H5 antibody and cells (non-permeabilized) grown at 30°C for 4 days in order to determine if ACE_Q1069R_ is misfolded. The proportion of i2H5^+^ cells in the population of ACE_Q1069R_ cells grown at 30°C increased 9-fold in contrast to cells grown at 37°C (Figure 4A); WT ACE was not affected (Figure 4A). We confirmed these results by fluorescence microscopy with ACE_Q1069R_ and ACE_WT_ cells grown at 30°C for 4 days. ACE_Q1069R_ cells grown at 30°C had more positively stained cells compared to cells grown at 37°C (Figure 4B). We examined ACE protein levels at the two different temperatures in order to exclude the possibility that the rescue of mutant ACE localization to the plasma membrane at 30°C was due to alteration in protein levels. Cells grown at 30°C did not affect protein levels of either WT or mutant proteins as compared to 37°C (Figure S4). Since growing cells at 30°C can augment the amount of the mutant protein present at the plasma membrane and this effect is not increased in the presence of proteasome inhibitor, we suspected that protein trafficking from the ER to Golgi was rescued at 30°C. To test this, HEK cells stably expressing ACE_Q1069R_ or ACE_WT_ were permeabilized and stained with anti-ACE i2H5 antibody and markers for calnexin and giantin; cells were then visualized by confocal microscopy (Figure 4C and Figure S5). Interestingly, there was an increase in co-localization of ACE_Q1069R_ with the Golgi, suggesting an improvement in ACE protein trafficking from the ER to Golgi (Figure 4C and Figure S5) resulting in partially restored surface expression of the mutant protein. Our results suggest that the point mutation in ACE gene is responsible for protein misfolding and retention in the ER leading to its degradation by the UPS; moreover, this defect can be partially overcome by growing cells at 30°C. To further confirm our findings we blocked protein trafficking through the secretory pathway using Brefeldin A (BFA) and tunicamycin. BFA interferes with protein transport from the ER to the Golgi apparatus, while retrograde transport from the Golgi to the ER stays unaffected, resulting in the partial redistribution of the Golgi resident protein to the ER. Furthermore, BFA also affects Golgi stacking, leading to dispersion of the Golgi; as a consequence, proteins are unable to reach the plasma membrane. Tunicamycin is an inhibitor of N-linked protein glycosylation affecting protein folding in the ER, which leads to an increase in ER protein retention time. If rescue at 30°C is indeed due to facilitated trafficking through the secretory pathway, treatment of cells with these drugs should oppose the rescue effect. Indeed, treatment of ACE_Q1069R_ cells with BFA and tunicamycin at 30°C, resulted in 50% lower protein surface expression as observed by immunofluorescence (Figure 5A). Moreover, tunicamycin decreased ACE_Q1069R_ colocalization with Golgi markers while BFA showed no colocalization and caused dispersion of the organelle (Figure 5B). Our results indicate that impaired trafficking through the secretory pathway causes reduced plasma membrane levels of ACE_Q1069R_ protein.

### Enzymatic Activity of ACE_Q1069R_

**Figure 6:**
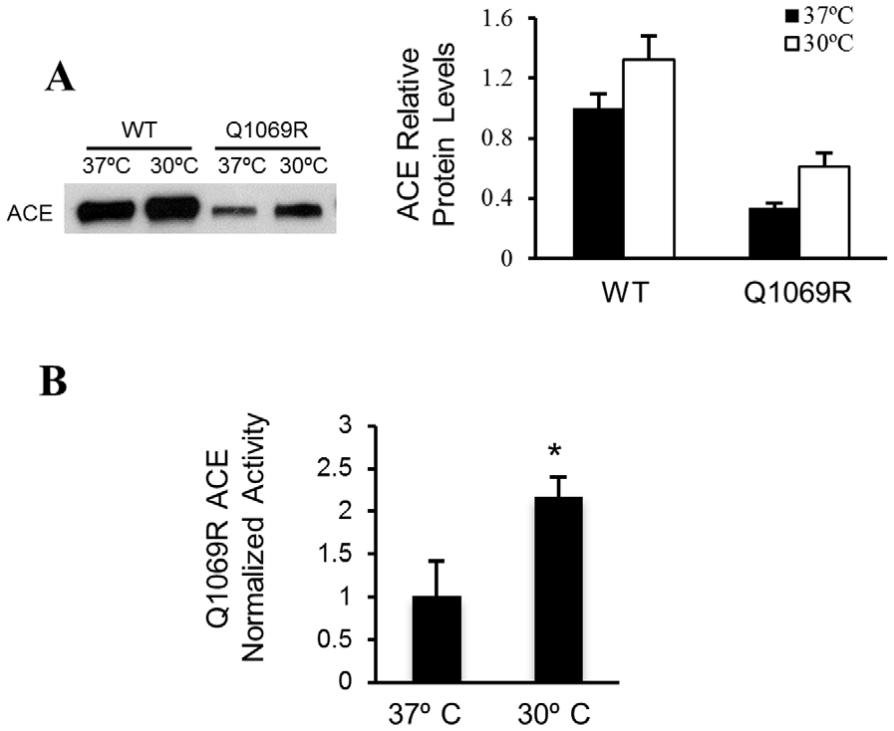
ACE_Q1069R_ activity is normal in HEK cells at 30°C. **A.** Western blot analyses of media from ACE_WT_ and ACE_Q1069R_ HEK cells grown at 37°C and 30°C. Equal amounts of total protein were loaded. Quantification of 3 independent experiments. Error bars represent ±SD. P<0.05. **B.** ACE activity in the media from ACE_Q1069R_ HEK cells grown at 37°C and 30°C. Activity was determined with a synthetic substrate (HHL) for 15 minutes incubation Activity was normalized by the amount of protein present in the media (quantification in A, using arbitrary units).

ACE_Q1069R_ is not able to reach its proper localization at the plasma membrane due to impaired trafficking (Figure 3A and D). We hypothesized that, once ACE_Q1069R_ reaches the cell surface, the mutant protein will be secreted into the extracellular fluids and its activity should be restored; this hypothesis is further supported by the modeling of the 3-D structure of the ACE protein, which suggests that the ACE_Q1069R_ point mutation should not affect the enzymatic activity of the protein (Figure 1B). To directly test our hypothesis, media from ACE_Q1069R_ and ACE_WT_ cells grown at 30°C for 4 days were harvested and tested by western blot and for ACE activity (Figure 6A and B). Interestingly, in the ACE_Q1069R_ cells ACE activity doubled when cells were grown at 30°C (Figure 6B), indicating that higher ACE levels at the plasma membrane and in the media is coupled with increased enzymatic activity in the media. In summary, our results suggest that if the ACE_Q1069R_ mutant protein reaches its proper localization in the cell, it will be secreted to the extracellular fluids where it can perform its normal function.

## Discussion

The correct expression, folding, trafficking and processing of proteins is essential for the overall cellular homeostasis and is emerging as a novel concept known as proteostasis [19]. However, the precise and intricate ways through cells regulate proteostasis are not fully understood, complicating the development of therapeutic strategies for several human disorders. Importantly, a recent study highlighted the potential of proteostasis modulation for the treatment of human diseases, showing that guanabenz, a small molecule used in hypertension treatments, rescued cells from protein misfolding stress due to elevated chaperone availability leading to improved folding [20].

We identified a novel mutation in the ACE gene responsible for autosomal RTD, a disorder that usually results in premature death, and characterized the molecular defects associated with the identified mutation.

A major challenge in the study of RTD was overcome by the development of an in vitro model which enabled us to demonstrate that a single amino acid substitution in the C-terminal domain of ACE causes the retention of the protein in the ER, degradation by the UPS, and altered trafficking, resulting in decreased presence on the plasma membrane. The trafficking of proteins from the ER is regulated by several critical and distinct mechanisms. First, quality control pathways ensure that only properly folded proteins are allowed to exit the ER [21]. Chaperone proteins within the ER participate in both protein folding and the retention of misfolded proteins within the ER [22]. The proteins that fail quality control are eliminated via the UPS system, in a process known as ERAD [23]. Thus, one possibility is that ACE_Q1069R_ protein misfolds due to the substitution of a neutral amino acid by a positively charged residue, altering folding kinetics. Both protein modeling and the observation that mutated ACE is present in low levels in the cell and is degraded by the proteasome support this theory. Alternatively, since ACE is composed of two similar domains, it may form intermediate dimers that are transport-competent to leave the ER and travel to the Golgi. Analysis of the quaternary structure of several membrane and secreted proteins supports the idea that dimer or oligomer formation is an important event that affects the rate of transport of proteins from the ER to the Golgi [24], [25].

Several disorders are caused the inability of a mutant protein to properly travel through the secretory pathway [26] including bilateral frontoparietal polymicrogyria and cystic fibrosis [27], [28]. In cystic fibrosis, the most common mutation in cystic fibrosis transmembrane conductance regulator (CFTR) leads to its retention in the ER and degradation by the UPS [29]; interfering with chaperone activity can prevent ER-sequestration, resulting in restored transport to the plasma membrane [30]. Incubation of cells at low temperature can have similar biological effects in cells with mutant CFTR, where low temperature enables the proper folding and maturation of the protein [31]. Similarly, localization of ACE at the plasma membrane was restored when we modulated the proteostasis network by growing cells at a lower temperature. Under these conditions the catalytic activity of the mutant protein was also restored suggesting that the mutation did not directly affect protein activity. When traffic through the secretory pathway was inhibited by BFA and tunicamycin, the mutant protein was unable to reach plasma membrane and no activity in the media was detected, confirming that the functional defect in mutant ACE activity was a consequence of aberrant protein trafficking rather than the loss of enzymatic activity.

Recently, it was shown that elevated levels of ACE expression is a risk factor in several cardiovascular and renal diseases and that overexpression of ACE is observed in breast, lung, and gastric cancer [32], [33], [34]; therefore, understanding the mechanism of ACE regulation is an important goal not only for the basic understanding of ACE biology, but also for clinical practice. Our work has several important consequences: first, it provides important insights into the functional structure of ACE; and secondly, it provides new information about the mechanism through which ACE mutations can lead to the severe cases of RTD.

## Materials and Methods

### Constructs and Cell Lines

HEK cells were a king gift from Dr. Luis Moita. Cells were in DMM supplemented with 10% heat inactivated fetal bovine serum, penicillin, streptomycin and L-Glutamine.

ACE_Q1069R_ was generated by site directed mutagenesis from the pCDNA3.1-Hygro-ACE_WT_ (kindly provided by Dr. Sergei Danilov). Site directed mutagenesis was performed as recommended with the QuickChange XL Site Directed Mutagenesis Kit (Stratagene). The CAG codon (glutamine) was mutated to a CGG codon (arg) at position 1069 using the forward primer: 5′-ggctgaagtaccggggcctctgccc-3′ and the reverse primer: 5′-gggcagaggccccggtacttcagcc-3′. The resulting construct was verified by sequencing.

### Immunoblot and Antibodies

Cells were washed with cold PBS, and lysed with NP-40 buffer in the presence of protease inhibitor cocktail (Roche). Lysates were cleared from debris by a 14K rpm centrifugation for 10 min at 4°C; total protein was quantified by Bradford Assay Kit (Biorad). Equal amounts of total protein were subjected to SDS-PAGE using 10% Tris–Glycine gel. Proteins on the SDS-PAGE were transferred to PVDF membranes (Biorad) and blocked in blocking buffer (5% milk in TBS with 0.1% Tween-20: TBS-T, pH 7.4) for 1h prior to the addition of the primary antibody overnight at 4°C. Primary antibodies: anti-ACE 1∶1000 (1D8) and anti-GAPDH 1∶5000 dilution (Ambion). Blots were washed three times with TBS-T and incubated at room temperature for 1h in HRP labeled secondary antibodies (GE Healthcare, 1∶10000 dilution). After three washes with TBS-T, immunoblots were developed using ECL (Millipore). The films were scanned and quantified with ImageJ.

### Molecular dynamics simulations

All simulations were performed with the GROMACS package and the all atom GROMOS96 force field. Presence of pocket and cavities within the structure were analyzed by POCASA algorithm. The atomic coordinates of ACE C-terminal domain from testis (PDB code: 2OC2) were used as initial model for the simulations. All the simulations were started with the aminoacid side chain conformations extracted from the PDB database with a protonation state consistent with a neutral pH. Proteins were solvated in a water box of 110 Å^3^ and a density of 1 g/cm3. The solvated models were energy minimized by conjugated gradient for 1000 steps to eliminate steric clashes between atoms. All the systems were equilibrated by simulated annealing with slow temperature decreasing from 2500 K to 300 K over 1000 cycles. Molecular dynamics simulations were then performed over 400 ps at 300 K and data collected every 1 ps.

### Flow cytometry analysis

HEK cells stably expressing ACE_WT_ and ACE_Q1069R_ were washed with ice cold PBS, detached with 1mM EDTA in PBS, resuspended in 0.5% BSA in PBS and stained with primary antibody (i2H5) for 20 min. After washing cells, cells were incubated with secondary antibody, alexa 488 anti-mouse, for 20 min. Acquisition was done on a FACS Calibur cytometer and analysis was performed with the FlowJo software.

### Immunocytochemistry and Fluorescence microscopy

Cells were plated in 12-well plates on a glass coverslip coated with poly-d-lysine (Sigma), grown at the corresponding temperatures (37°C or 30°C). We added Brefeldin A at 10 µg/ml for 6h and tunicamycin at 10 µg/ml for 6 h. Cells were fixed in 4%PFA for 10 min, permeabilized with 0.5% Triton-100 in TBS and blocked for 1h with 1.5% goat serum. Primary antibodies were: anti-ACE 2iH5, anti-Calnexin antibody (1∶100 dilution; Invitrogen), and giantin (1∶250 dilution, ABCAM). Alexa 488 and Alexa 568 conjugated secondary antibodies were used at a 1∶1000 dilution. Coverslips were mounted with Vectashield and visualized on a Zeiss LSM 510 META confocal microscope using a 63x/1.4 oil immersion objective. Sequential multi-track frames were acquired to avoid any potential crosstalk from the two fluorophores. Quantification of co-localization was performed in ImageJ (http://rsbweb.nih.gov/ij/). For details see SI.

### Real time PCR

Total RNA was extracted using Trizol reagent according to the manufacturer (Invitrogen). cDNA was synthesized from 4 µg total RNA using random primers and superscript II reverse transcriptase following the manufacturer instructions (Invitrogen). Real time PCR was performed using SYBR green master mix according to the manufacturer manual (Sigma Aldrich). All reactions were performed in triplicates. PCR primers were specific for ACE (GGT GGT GTG GAA CGA GTA TG, TCG GGT AAA ACT GGA GGA TG) and β-actin. Quantification was done in following the Pfaffl method.

### *In vivo* Protein Stability Assay

HEK cells stably expressing ACE_WT_ or ACE_Q1069R_ were grown in the presence of 10uM Cyclohexamide (CHX) and either ethanol or 10uM ALLN (N-acetyl-L-leucyl-L-leucyl-L-norleucine) (both from Sigma), cells were harvest at each time point and lysates were analyzed by western blot. The films were scanned and quantified with ImageJ.

### Enzymatic Activity

Media in which stable cells were grown was collected (10ml per 10cm dish) and concentrated using a Vivaspin-15R column following manufacture instruction (Sartorius stedim). BCA was used to quantify total protein in 10X concentrated media. For ACE activity in the blood, a peripheral blood sample using standard procedures was extracted from patient and 5 healthy volunteers. Written informed consent was obtained from all of the participants according to the Declaration of Helsinki, and the study was approved by the ethics committee of the Hospital de Santa Maria, Lisbon, Portugal. ACE colorimetric enzymatic assay was performed according to the manufacturer (Buhlmann). Briefly, equal amount of total protein from the media were added to 300 µl of incubation buffer and 200 µl substrate (HHL) for 15 min at 37°C. The product was quantified by measuring emission wavelength at 382nm.

### Sequencing

Genomic DNA of the patient, his parents and brother were extracted from a peripheral blood sample using standard procedures. Written informed consent was obtained from all of the participants according to the Declaration of Helsinki, and the study was approved by the ethics committee of the Hospital de Santa Maria, Lisbon, Portugal. The ACE gene was analyzed for mutations. Intronic primers that flank each of the 26 ACE exons were used (GenBank accession number NM_000789) for sequencing. PCRs were performed with 100 ng template of genomic DNA denatured for 5 min at 94^o^C followed by 30 cycles of amplification (45s at 95^o^C; 45s at specific annealing temperature; 45 s at 72°C) followed by a 10 min extension of 72°C. PCR products were tested on a 2% agarose gel. PCR products were sequenced on an automated sequencer ABI PRISMR 3100-Avant using a BigDye v3.1 sequence kit (Applied Biosystems) and analysis was done on both strands of the PCR amplified exons whenever a mutation was found.

## Supporting Information

Figure S1 ACE activity in the plasma from the RTD patient and 5 controls healthy volunteers (marked 1 to 5) using a synthetic substrate (HHL) for15 minutes incubation.
(TIF)

Figure S2 Evolution of average RMSD of each aminoacid and overall hydrodynamic properties of ACE C-terminal domain wild type and Q1069R mutant during the moleculardynamics simulation. Parameters along the simulation were represented by black lines for ACE WT protein and by red lines for ACE Q1069Rmutant. Panels A: aminoacid average RMSD along the simulation. The upper scheme represents the relative position of the aminoacids belonging to the active site of the enzyme and involved in the coordination of the Zn2+atom and the location of the characterized Q1069R mutation; Panels A: hydrophobic solvent accessible surface; B: hydrophobic solvent accessible surface; C: hydrophilic solvent accessible surface; D: total solvent accessible surface; E: Rx hydrodynamic radius; F: Ry hydrodynamic radius and G: Rz hydrodynamic radius.
(TIF)

Figure S3 Immunostain analysis by confocal microscopy of ACE_Q1069R_ and ACE_WT_ cells grown at 37°C on glass coverslips. Cell were fixed and permeabilized, then double stained for ACE (green) and Golgi (red). Nuclei are stained blue with DAPI. Inset represent enlarged images in the boxed regions. Scale bars, 20 µm. Quantification of the green signal overlapping with red signal was done using ImageJ software. Quantification of 3 independent experiments. Error bars represent ±SD. P<0.05.
(TIF)

Figure S4 Western blot analysis of ACE protein levels in ACE_Q1069R_ and ACE_WT_ cells. Cells were grown at 37°C or 30°C for 4 days. Quantification of 3 independent experiments. Error bars represent ±SD.
(TIF)

Figure S5 Immunostaining analysis by confocal microscopy of ACE_Q1069R_ cells grown at 37°C or 30°C for 4 days on glass coverslips. Cell were fixed and permeabilized, then double stained for ACE (green) and ER (red). Nuclei are stained blue with DAPI. Inset represents enlarged images in the boxed regions. Scale bars, 20 µm.
(TIF)
