# Supplementary figures and images for: Impaired Proteostasis Contributes to Renal Tubular Dysgenesis
Source: PLoS One. 2011 Jun 9;6(6):e20854. doi: 10.1371/journal.pone.0020854 (PMC3111453; doi:10.1371/journal.pone.0020854)

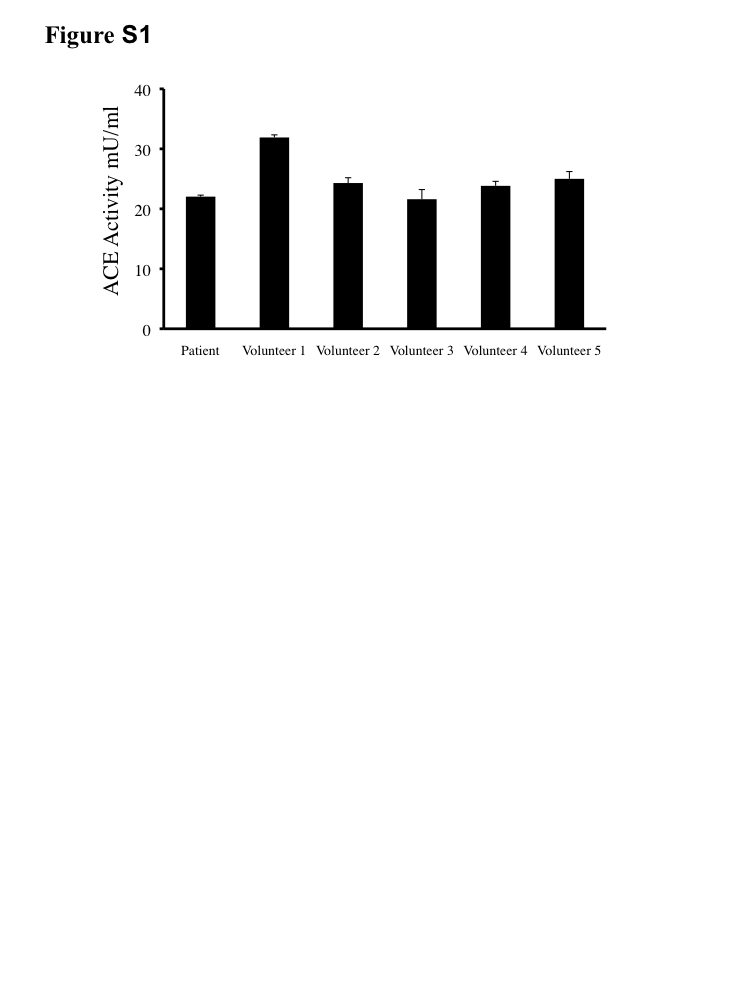

Supplement: Figure S1 — ACE activity in the plasma from the RTD patient and 5 controls healthy volunteers (marked 1 to 5) using a synthetic substrate (HHL) for15 minutes incubation. (TIF) [file pone.0020854.s001.tif]

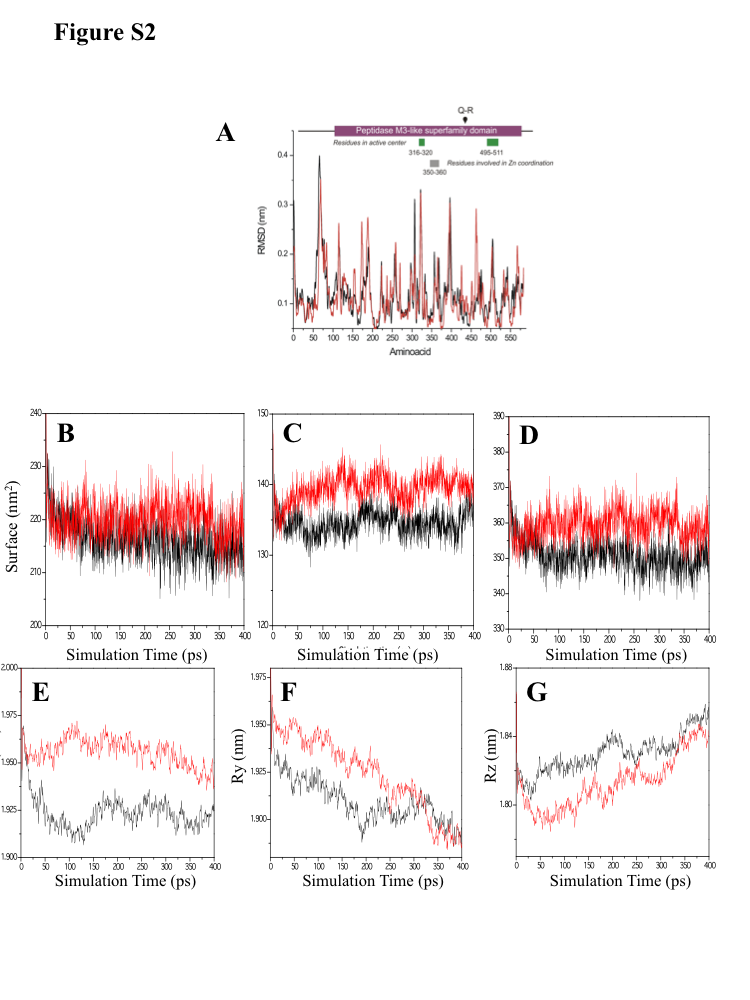

Supplement: Figure S2 — Evolution of average RMSD of each aminoacid and overall hydrodynamic properties of ACE C-terminal domain wild type and Q1069R mutant during the moleculardynamics simulation. Parameters along the simulation were represented by black lines for ACE WT protein and by red lines for ACE Q1069Rmutant. Panels A: aminoacid average RMSD along the simulation. The upper scheme represents the relative position of the aminoacids belonging to the active site of the enzyme and involved in the coordination of the Zn2+atom and the location of the characterized Q1069R mutation; Panels A: hydrophobic solvent accessible surface; B: hydrophobic solvent accessible surface; C: hydrophilic solvent accessible surface; D: total solvent accessible surface; E: Rx hydrodynamic radius; F: Ry hydrodynamic radius and G: Rz hydrodynamic radius. (TIF) [file pone.0020854.s002.tif]

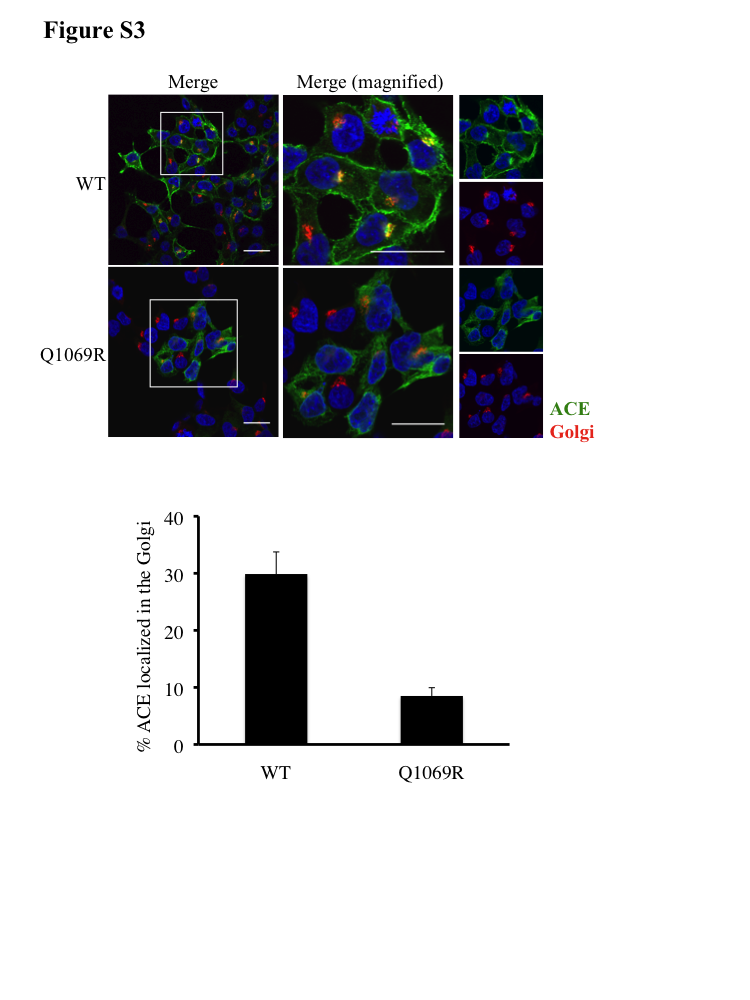

Supplement: Figure S3 — Immunostain analysis by confocal microscopy of ACEQ1069R and ACEWT cells grown at 37°C on glass coverslips. Cell were fixed and permeabilized, then double stained for ACE (green) and Golgi (red). Nuclei are stained blue with DAPI. Inset represent enlarged images in the boxed regions. Scale bars, 20 µm. Quantification of the green signal overlapping with red signal was done using ImageJ software. Quantification of 3 independent experiments. Error bars represent ±SD. P<0.05. (TIF) [file pone.0020854.s003.tif]

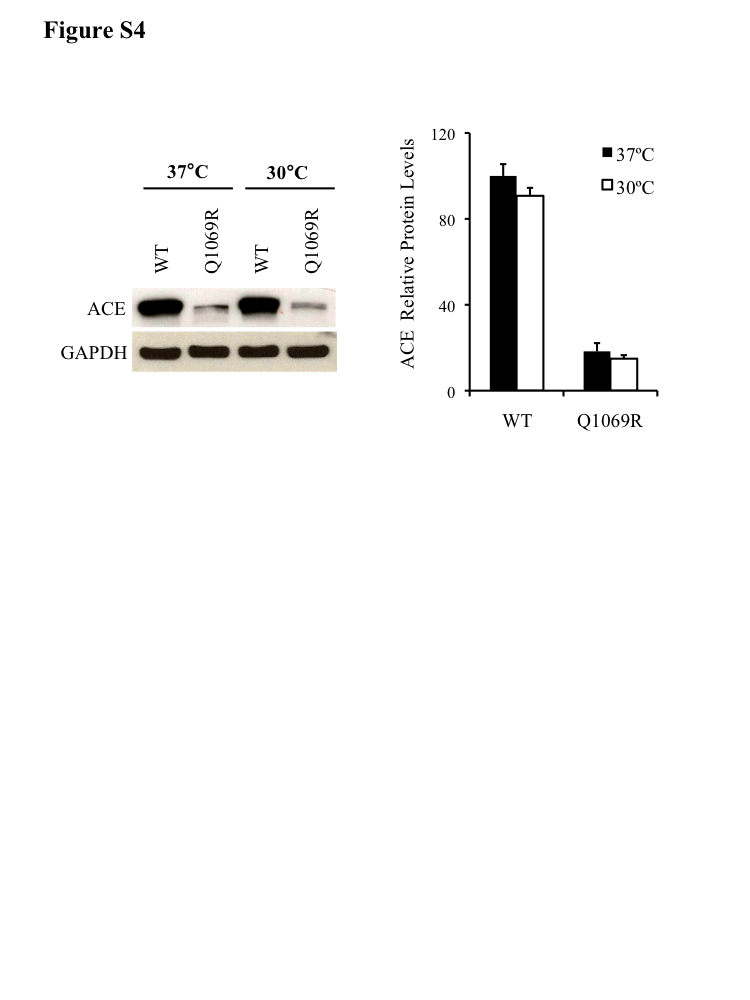

Supplement: Figure S4 — Western blot analysis of ACE protein levels in ACEQ1069R and ACEWT cells. Cells were grown at 37°C or 30°C for 4 days. Quantification of 3 independent experiments. Error bars represent ±SD. (TIF) [file pone.0020854.s004.tif]

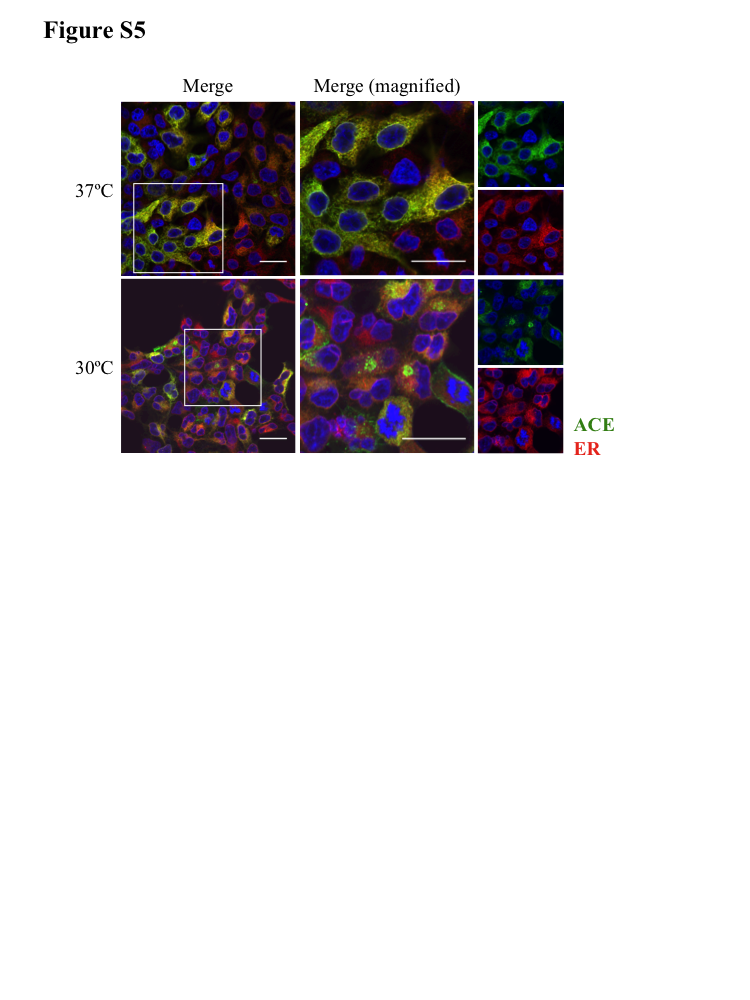

Supplement: Figure S5 — Immunostaining analysis by confocal microscopy of ACEQ1069R cells grown at 37°C or 30°C for 4 days on glass coverslips. Cell were fixed and permeabilized, then double stained for ACE (green) and ER (red). Nuclei are stained blue with DAPI. Inset represents enlarged images in the boxed regions. Scale bars, 20 µm. (TIF) [file pone.0020854.s005.tif]
